# Anidulafungin for candidemia/invasive candidiasis in non-neutropenic ICU patients

**DOI:** 10.1186/cc9660

**Published:** 2011-03-11

**Authors:** M Ruhnke, J Paiva, W Meersseman, J Pachl, I Grigoras, G Sganga, F Menichetti, P Montravers, G Auzinger, G Dimopoulos, M Borges Sá, P Miller, T Marček, M Kantecki

**Affiliations:** 1 Charité University Hospital, Berlin, Germany; 2 Hospital São João, Porto, Portugal; 3 University Hospital Leuven, Belgium; 4 University Hospital Královské Vinohrady, Prague, Czech Republic; 5 University Hospital Sf Spiridon, Iaśi, Romania; 6 University Hospital A Gemelli, Rome, Italy; 7 University Hospital Pisa, Italy; 8 Hospital Bichat Claude Bernard, Paris, France; 9 King's College Hospital, London, UK; 10 University Hospital Attikon, Haidari, Greece; 11 Hospital Son Llatzer, Palma de Mallorca, Spain; 12 Pfizer, Sandwich, UK; 13 Pfizer, Paris, France

## Introduction

A recent study found anidulafungin (ANI) safe and effective for candidemia/invasive candidiasis (C/IC) in selected populations of ICU patients [1]. A *post hoc *analysis of this study was performed to evaluate the efficacy of ANI in the same populations, but in non-neutropenic C/IC patients only.

## Methods

A prospective, open label, multinational, phase 3b study in adult ICU patients (APACHE II score <25) with ≥1 of the following: post-abdominal surgery; age ≥65 years; renal/hepatic insufficiency; solid organ transplant; neutropenia; and/or solid tumor. C/IC was confirmed from 96 hours before to 48 hours after the start of study treatment. Patients received i.v. ANI (200 mg on day 1, 100 mg/day thereafter) for ≥10 days, with optional oral azole step-down therapy, for a total treatment duration of 14 to 56 days. Primary efficacy endpoint was global response at end of all therapy (EOT) in the evaluable modified intent-to-treat (eMITT) population; that is, excluding patients with missing/unknown responses. For the present analysis, all patients with neutropenia were excluded.

## Results

The total MITT population (that is, confirmed C/IC and ≥1 dose of ANI) included 170 patients, 157 (92.4%) of whom were non-neutropenic. In these patients at baseline, 69.4% had candidemia, mean APACHE II score was 16.3 (range 4 to 26) and mean SOFA score 7.4 (range 0 to 20). In non-neutropenic eMITT patients, global response at EOT was 71.1% (95% CI = 62.9, 78.4). At the end of i.v. therapy, 2 weeks post-EOT and 6 weeks post-EOT the global response was 72.4%, 61.2% and 52.0%, respectively. When missing/unknown responses were included and classed as failures, global success was 64.3% at EOT. The 90-day Kaplan-Meier survival estimate was 55.0% (95% CI = 47.2, 62.9). Among all non-neutropenic patients with ≥1 dose of ANI, treatment-related (due to ANI and/or azole) AEs and serious AEs occurred in 29/201 (14.4%) and 3/201 (1.5%) of patients, respectively. The most common treatment-related AE was erythema in four patients (2.0%). Other treatment-related AEs occurred in ≤1.5% of non-neutropenic patients.

## Conclusions

ANI was effective and safe for the treatment of C/IC in selected populations of non-neutropenic ICU patients.
